# Daily Fructose Traces Intake and Liver Injury in Children with Hereditary Fructose Intolerance

**DOI:** 10.3390/nu11102397

**Published:** 2019-10-07

**Authors:** Fabiola Di Dato, Simona Spadarella, Maria Giovanna Puoti, Maria Grazia Caprio, Severo Pagliardini, Claudia Zuppaldi, Gianfranco Vallone, Simona Fecarotta, Gabriella Esposito, Raffaele Iorio, Giancarlo Parenti, Maria Immacolata Spagnuolo

**Affiliations:** 1 Department of Translational Medical Science, Section of Pediatrics, University of Naples Federico II, 80131 Naples, Italy; fabiola.didato@unina.it (F.D.D.); s.spadarella@gmail.com (S.S.); claudiazuppaldi@libero.it (C.Z.); simona.fecarotta@gmail.com (S.F.); parenti@unina.it (G.P.); mispagnu@unina.it (M.I.S.); 2 Italian Society of Pediatric Gastroenterology and Nutrition (SIGENP), 20126 Milan, Italy; 3 Great Ormond Street Hospital for Children NHS Foundation Trust, Great Ormond St., Holborn, London WC1N 3JH, UK; giovanna.puoti@hotmail.it; 4 Institute of Biostructure and Bioimaging National Research Council, 80145 Naples, Italy; mariagrazia.caprio@ibb.cnr.it; 5 Department of Pediatrics and Public Health and Pediatric Sciences, University of Torino, 10126 Torino, Italy; pagliardini.severo@virgilio.it; 6 Pediatric Radiology, Department of Advanced Biomedical Sciences, University of Naples Federico II, 80131 Naples, Italy; gianfranco.vallone1@gmail.com; 7 Department of Molecular Medicine and Medical Biotechnology, University of Naples Federico II, 80131 Naples, Italy; gabriella.esposito@unina.it; 8 CEINGE Advanced Biotechnologies, 80145 Naples, Italy; 9 Telethon Institute of Genetics and Medicine, 80078 Pozzuoli, Italy

**Keywords:** Hereditary fructose intolerance, fructose, sucrose, sorbitol, sialotransferrin profile, aldolase B, liver steatosis

## Abstract

Background: Hereditary fructose intolerance (HFI) is a rare genetic disorder of fructose metabolism due to aldolase B enzyme deficiency. Treatment consists of fructose, sorbitol, and sucrose (FSS)-free diet. We explore possible correlations between daily fructose traces intake and liver injury biomarkers on a long-term period, in a cohort of young patients affected by HFI. Methods: Patients’ clinical data and fructose daily intake were retrospectively collected. Correlations among fructose intake, serum alanine aminotransferase (ALT) level, carbohydrate-deficient transferrin (CDT) percentage, liver ultrasonography, genotype were analyzed. Results: We included 48 patients whose mean follow-up was 10.3 ± 5.6 years and fructose intake 169 ± 145.4 mg/day. Eighteen patients had persistently high ALT level, nine had abnormal CDT profile, 45 had signs of liver steatosis. Fructose intake did not correlate with ALT level nor with steatosis severity, whereas it correlated with disialotransferrin percentage (R^2^ 0.7, *p* < 0.0001) and tetrasialotransferrin/disialotransferrin ratio (R^2^ 0.5, *p* = 0.0001). p.A150P homozygous patients had lower ALT values at diagnosis than p.A175D variant homozygotes cases (58 ± 55 IU/L vs. 143 ± 90 IU/L, *p* = 0.01). Conclusion: A group of HFI patients on FSS-free diet presented persistent mild hypertransaminasemia which did not correlate with fructose intake. Genotypes may influence serum liver enzyme levels. CDT profile represents a good marker to assess FSS intake.

## 1. Introduction

Hereditary fructose intolerance (HFI) is a rare genetic disorder of fructose metabolism related to a deficiency of the aldolase B (ALDOB) and intracellular accumulation of toxic fructose 1-phosphate in the liver, intestine, and kidney [1]. Ingestion of fructose causes acute symptoms such as vomiting and diarrhea, but also life-threatening manifestations, including acute hypoglycemia, lethargy, renal tubular acidosis, and acute liver failure. Chronic ingestion of fructose results in poor feeding, failure to thrive or growth retardation, liver and kidney damage [2]. If diagnosed early and accurately treated, HFI is a preventable life-threatening condition, but without prompt intervention, it can be lethal [3]. To date, the only available therapy is a fructose, sorbitol, and sucrose (FSS)-free diet. Total exclusion of these sugars is not easily feasible because of small hidden fructose amounts contained in many foods. It is not clear whether small amounts of fructose can be tolerated and if a threshold exists, above which liver damage occurs. In previous reports, amounts of 40 mg/kg/day or less or 1.5 g/day were considered safe for HFI patients [4,5]. While it was reported that inadequate fructose restriction causes growth deficiency even in clinically asymptomatic HFI patients [4], possible long-term effects of chronic fructose traces ingestion are unknown.

Carbohydrate-deficient transferrin (CDT) profile, initially used as an additional tool for identification of HFI patients [6,7], was demonstrated to be abnormal even in some patients on FSS-free diet [8]. However, this aspect was not further investigated.

The principal aim of this study was to assess disease-related clinical and biochemical features in HFI patients on a long-term period of FSS-free diet. We evaluated possible correlations among residual fructose intake, liver function tests, CDT profile and genotype.

## 2. Materials and Methods

We retrospectively evaluated the clinical records of all HFI patients followed-up at the Translational Medical Science Department, Section of Pediatric, of University of Naples Federico II, Italy. The study was conducted in conformity with the Declaration of Helsinki.

HFI diagnosis was confirmed by the presence of *ALDOB* pathogenic variants on both alleles or by ALDOB activity assay in liver biopsy. Inclusion criteria were availability of clinical and biochemical data, and ultrasound parameters for at least four years.

In the first part of the study, clinical and auxological parameters, biochemical features of liver injury and liver ultrasound (US) findings were evaluated. We considered as onset of disease the beginning of symptoms in symptomatic patients and diagnosis time in patients whose diagnosis was made by family screening. We reviewed blood tests at diagnosis (T0) and at the following time points: 6 months (T1), 12 months (T2), 18 months (T3), 24 months (T4), 36 months (T5), 48 months (T6), and at the last evaluation (T7).

### 2.1. Clinical and Biochemical Parameters

Patients’ clinical data were retrospectively reviewed. At each time point, clinical history, symptoms (aversion to sweet food, poor feeding, vomiting, diarrhea, hypoglycemia-related symptoms, and failure to thrive), auxological parameters (weight, height, weight/height ratio), physical examination, neuro-psychomotor development, and blood laboratory tests including alanine aminotransferase (ALT), gamma-glutamyltransferase (γ-GT), albumin, total proteins, total bilirubin, glucose blood level, cholesterol, international normalized ratio (INR), serum creatinine, and urea were evaluated. Laboratory tests were performed using standard methods.

Based on ALT values during follow-up, patients were classified into two groups: “with hyperALT” (H-group) and “without hyperALT” (nH-group). We defined as hyperALT an increase in ALT level of at least 1.5 folds the upper limit (40 U/L) of the normal range, more than three times during observation. In H-group patients, other causes of liver disease such as obesity, coeliac disease, drug-related toxicity, infections, autoimmune hepatitis, biliary system disease, Wilson disease, cystic fibrosis, and extra-hepatic causes of hypertransaminasemia (i.e., muscle diseases) were ruled out.

### 2.2. Ultrasound Evaluation

The images were obtained with General Electric (GE) Logiq 9 ultrasound machine using a multifrequency convex transducer (4 MHz). An expert pediatric radiologist carried out US. Three parameters, including parenchyma echogenicity, far gain attenuation, and diaphragm blurring, were assessed by qualitative evaluation comparing the echogenicity of the hepatic parenchyma with that of right kidney cortical. The radiologist graded US examination according to the presence and severity of liver steatosis by using the following criteria:

1. Normal liver echotexture (absence of steatosis).
2. Mild hyperechoic liver tissue (compared with the adjacent kidney cortex) was considered as mild steatosis.
3. Diffuse increase of echo intensity with decreased beam penetration (with slightly decreased visualization of the diaphragm) associated with a decrease in the visualization of silhouetting of the portal vein borders was considered as moderate steatosis.
4. The marked increase in echoes intensity with no visualization of portal vein border, obscured diaphragm and posterior portion of the right lobe was considered as severe steatosis.

A quantitative assessment of the presence of steatosis was performed to support qualitative evaluation, sampling regions of interest (ROI) on the right hepatic lobe and on the renal cortex. Three ROIs were drawn on the right lobe of the liver at the same depth as that of the kidney and in each ROI, a numerical value measured in decibels (dB) was calculated. It expresses the attenuation of the ultrasound beam in the studied area. This value is inversely proportional to the degree of echogenicity and correlates with the steatosis degree. The mean ROI value of the right kidney was −57.4 ± 4.5 dB and consequently right hepatic lobe ROI values higher than −50 dB, which was considered as indicative of liver steatosis. The average value of the ROIs in the right hepatic lobe was evaluated for each patient. Based on the qualitative assessment of steatosis degree, patients were divided into four groups: (1) Absence of steatosis, (2) mild steatosis, (3) moderate steatosis, (4) severe steatosis. The average value of the ROIs was calculated and compared for each subgroup.

### 2.3. Assessment of Diet

The diet was evaluated with a seven-day food diary kept at home by adult patients, by patients over 12 with the help of parents and only by parents for younger patients. In the food diary, collected for each patient at the time of the last observation, each food and its quantity were recorded. An expert pediatric dietician in metabolic diseases instructed the patients and their parents how to fill out the food diary, reviewed the collected data and calculated the FSS intake. The amount of sugars introduced through diet was expressed in mg/day. Correlation between FSS intake and ALT levels measured at the same time was analyzed.

### 2.4. Transferrin Isoelectric Focusing

Blood samples were placed on specialized filter paper, dried at room temperature, and sent to a laboratory. Glycosylation pattern of serum sialotransferrin was analyzed by isoelectric focusing (IF). In particular, transferrin isoforms were separated by electrophoresis on 1% agarose gel (Iso-Gel agarose, FMC) with 5% ampholine in a pH range of 5.0–7.0 on a Multiphore 2117 apparatus (LKB) with a modified electrode lid. After IF, transferrin isoforms were visualized by immunofixation and a qualitative assessment of CDT was obtained. A quantitative analysis of disialotransferrin (DST) and tetrasialotransferrin (TST) was determined by estimating migration bands through Image J program. Transferrin serum isoforms were expressed as disialotrasferrin (DST) and tetrasialotransferrin (TST) percentage. Tetrasialotransferrin/disialotransferrin ratio (TST/DST) was assessed. We correlated FSS intake with DST% and TST/DST, respectively.

### 2.5. ALDOB Molecular Analysis and Genotype-Phenotype Correlation

Genomic DNA was extracted from peripheral blood samples collected in ethylenediaminetetraacetic acid, according to standard protocol. The *ALDOB* promoter region and the eight coding exons with the splice sites were amplified by polymerase chain reaction and analyzed by Sanger sequencing [9].

To assess the impact of *ALDOB* pathogenic sequence variants on the patients’ phenotype, the following variables were compared between different groups of genotypes: age at diagnosis, aversion to sweet taste, ALT levels at diagnosis and during follow-up.

### 2.6. Statistical Analysis

Continuous variables with normal distribution were presented as the number of patients (N), means and standard deviation (SD) and compared by using Student’s *t*-test, multiple *t*-tests. Discrete variables were presented as percentage (%), median and range and χ^2^ test (or Fisher’s exact test if necessary) were used to compare groups of patients. Linear regression was used to correlate continuous variables. All *p*-values were based on the one-tailed or two-tailed comparisons as appropriate and those less than 0.05 were considered to indicate a statistically significant difference. Statistical analysis was performed using SPSS for IOS software version 22.0 (IBM Corp, Armonk, NY, USA).

## 3. Results

According to the inclusion criteria, forty-eight patients, from 39 unrelated families, were studied (23 males, mean age 12.8 ± 5.7 years). The mean age at diagnosis was 2.5 ± 3.6 years, mean follow-up was 10.3 ± 5.6 years. In 47 patients, HFI was confirmed by the presence of *ALDOB* pathogenic variants on both alleles, in one patient, deficiency of ALDOB was assessed by liver biopsy sampling.

### 3.1. Clinical and Biochemical Parameters

The most frequent symptom at onset was an aversion to sweet taste reported in 25 patients (52%). Five out of seven patients detected by familial screening were asymptomatic. Clinical findings are shown in Table 1.

No signs of growth deficiency were found in all patients both at diagnosis and at the end of the evaluation, in terms of weight and height. The median weight–height ratio was 50th centile (range 5–95) at the diagnosis and 50th centile (range 5–95) at the end of follow-up. During follow-up, none of the patients required hospitalization for severe symptoms related to HFI. No patient showed signs of neurological or intellectual impairment.

Signs of liver disease, such as hypertransaminasemia and/or liver steatosis on US, were found at onset in 36 (75%) patients (Table 1). Among the seven patients detected by familial screening, five had US signs of liver steatosis at diagnosis associated in two cases with hypertransaminasemia, whereas two had no signs of liver disease.

**Figure 1:**
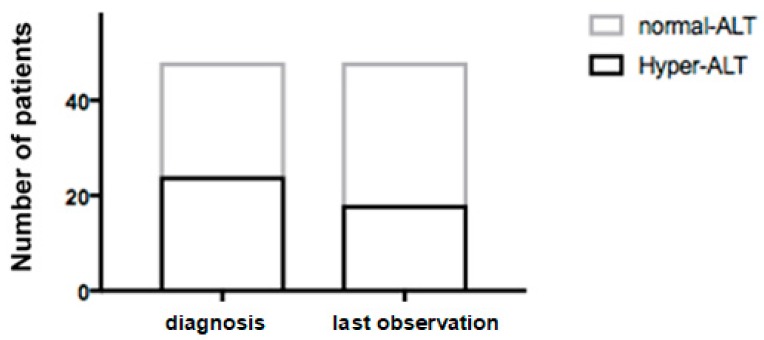
HFI patients (*n* = 48) with and without hypertransaminasemia at diagnosis and at the end of follow-up. ALT, alanine aminotransferase.

Twenty-four (50%) patients presented hypertransaminasemia at diagnosis (ALT 139 ± 104 IU/L) and 24 had normal ALT values (26 ± 9 IU/L) (Figure 1).

No significant difference was found in the age at diagnosis between these two groups (1.6 ± 1.3 vs. 3.3 ± 4.8 years). Other liver function tests, blood glucose levels, and renal function tests were normal in all patients at each time of evaluation. None showed signs of liver failure.

During follow-up, 18 (37.5%) of 48 patients showed persistently elevated ALT levels (H-group). The comparison of average ALT values at different time points between H-group and nH-group is shown in Table 2.

At the time of diagnosis, H-group patients had a higher ALT level compared to nH-group (117 ± 115 vs. 61 ± 60 IU/L; *p* = 0.02). No significant difference in other laboratory tests (Table 3), age at onset (2.1 ± 4.1 vs. 2.6 ± 3.2 years), age at evaluation (14 ± 5.8 vs. 12.1 ± 5.6 years), follow-up duration (11.8 ± 5.3 vs. 9.5 ± 5.4 years) and presence of sweet aversion (50% vs. 56.7%) was found between these two groups of patients.

### 3.2. Ultrasound Evaluation

Regarding US qualitative evaluation, 35 patients (72.9%) had signs of liver steatosis at diagnosis. At the end of follow-up, 45 (93.8%) patients presented liver steatosis. None showed signs of liver cirrhosis. At the end of follow-up, 14 patients (29.2%) had mild steatosis, 22 (45.8%) moderate and nine (18.5%) severe.

As for quantitative assessment, ROI data were obtained in 40/48 patients. A significant difference between the mean values of the ROIs was found in the right liver lobe in patients without and with mild steatosis (*p* = 0.001). The same occurred in patients with mild and moderate steatosis (*p* = 0.0003) and in patients with moderate and severe steatosis (*p* = 0.045).

### 3.3. Fructose Intake

FSS-free diet was evaluated in all patients. Based on weekly food diary, the average fructose intake was 169 ± 145.4 mg/day and none exceeded the recommended amount of 1.5 g/day. No significant difference in fructose intake was observed between H- and nH-group (164.8 ± 169.8 vs. 171.5 ± 131.6 mg/day) and there was no correlation between fructose intake and ALT levels.

### 3.4. Serum Sialotransferrin Isoelectric Focusing

**Figure 2:**
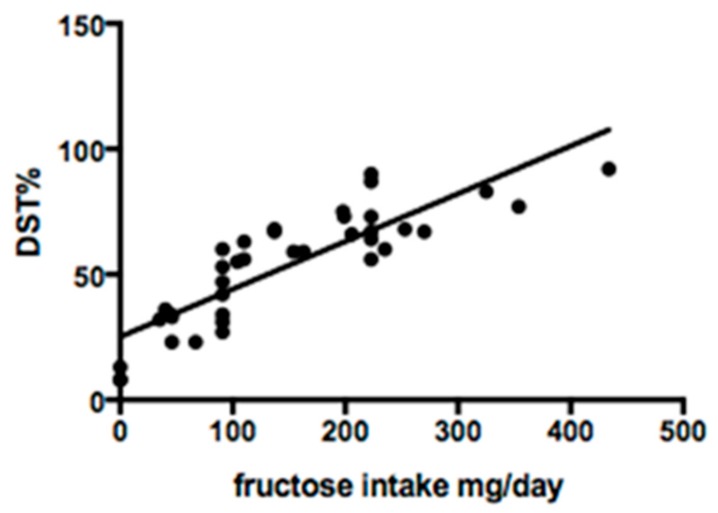
Linear regression between fructose intake and disialotransferrin (DST) %.

**Figure 3:**
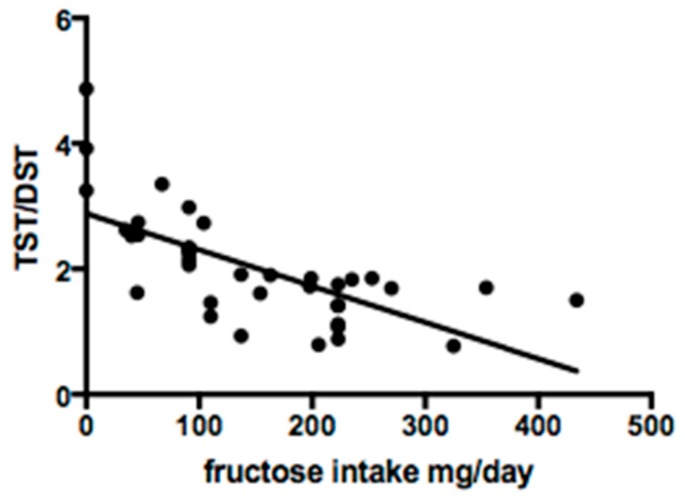
Linear regression between fructose intake and tetrasialotransferrin/disialotransferrin ratio (TST/DST).

Serum sialotransferrin isoforms were studied in 38 of 48 patients. Qualitative analysis showed a normal profile in 29/38 (76.3%) cases. Quantitative analysis revealed mean DST% of 53.4 ± 22.7, TST% 92.9 ± 33.4 and TST/DST ratio 2.0 ± 0.9. Fructose intake was significantly correlated with DST% (R^2^ 0.7; *p* < 0.0001) and TST/DST ratio (R^2^ 0.5; *p* = 0.0001) (Figure 2 and Figure 3).

### 3.5. Genotype Analysis

**Figure 4:**
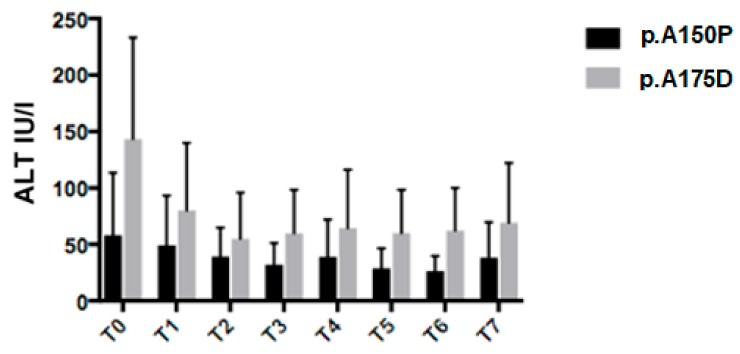
Alanine aminotransferase (ALT) values at different time points between p.A150P/p.A150P and P.A175D/P.A175D HFI patients. T0: time of diagnosis; T1: 6 months; T2: 12 months; T3: 18 months; T4: 24 months; T5: 36 months; T6: 48 months; T7: end of follow-up.

Nine different *ALDOB* pathogenic sequence variants were found in our cohort of HFI patients (Table 1). In accordance with previous reports [9,10], the most common variants were the missense p.A150P (44.8%), p.A175D (31.2%), and p.N335K (3.1%), the nonsense p.Y204* (11.4%), the frameshift p.N120Kfs* (3.1%). Twenty patients were homozygous for the most common variants, p.A150P and p.A175D, respectively, and therefore, were eligible for the genotype-phenotype assessment. We compared parameters of 14 p.A150P homozygous patients to six p.A175D homozygous patients. Average ALT values were lower in p.A150P/p.A150P patients than p.A175D/p.A175D, at diagnosis and during follow-up (U/L: T0 58 ± 55 vs. 143 ± 90, *p* = 0.01; T1 49 ± 44 vs. 80 ± 60, *p* = NS; T2 39 ± 26 vs. 55 ± 41, *p* = NS; T3 32 ± 18 vs. 60 ± 39, p = 0.04; T4 39 ± 33 vs. 65 ± 52, p = NS; T5 29 ± 18 vs. 60 ± 38, *p* = 0.02; T6 26 ± 13 vs. 62 ± 38, *p* = 0.004; T7 38 ± 31 vs. 69 ± 53, *p* = NS) (Figure 4).

These two groups did not differ in fructose intake (179.8 ± 150.1 vs. 94.8 ± 92.9 mg/day). No significant difference was found for age at onset and sweet food aversion.

### 3.6. Multivariate Analysis

Multiple regression analysis was used to develop a model for determining ALT values depending on multiple independent variables such as daily fructose intake, genotype, age, and sex. Only the genotype showed a significant impact in the full model (*p* = 0.003).

## 4. Discussion

HFI is an orphan metabolic disorder that clinically appears when fructose is introduced in the diet. Untreated patients develop liver, kidney, and intestinal damage with growth failure, but total exclusion of toxic sugars (i.e., fructose, sucrose and sorbitol) is not easily feasible because of their small hidden amounts contained in many foods.

Unfortunately, the maximum tolerable amount of these toxic sugars is not well defined, just as it is not well known whether the tolerance to them increases with age. In this study, we evaluated the amount of daily fructose intake and its clinical impact in our HFI patients on FSS-free diet. To evaluate mean fructose daily intake, we used a seven-day food diary completed by patients and/or parents. Obviously, a seven-day food diary cannot be representative of the patient’s entire medical history, however, it is considered as a reliable indicator of the patient’s current eating habits, as reported in the literature [11]. Furthermore, the correlation between liver function and fructose intake was assessed at the same time as the food diary was completed. Notably, all enrolled patients assumed less than 1.5 gm/day of FSS and showed good clinical conditions and normal auxological parameters after a 10-year mean follow-up. Nevertheless, the majority of them showed sonographic evidence of liver steatosis and one third had mild hypertransaminasemia. An increase in serum transaminases levels may be one of the reasons why undiagnosed HFI patients come to observation, especially in countries such as Italy where transaminases determination is routinely performed at check-up or before surgical procedures in absence of a clear clinical indication [12].

The rate and time of normalization of transaminases levels in patients with HFI adherent to FSS-free diet are not known. Interestingly, although fructose intake was lower than the recommended amounts for HFI [4,5], a subgroup of our patients presented persistent biochemical and ultrasound signs of mild liver injury. Particularly, 37.5% showed persistent high ALT values and no correlation was found between residual fructose intake and ALT values. Besides, 93.8% had sonographic signs of liver steatosis at the end of follow-up. Recently it was suggested that non-alcoholic fatty liver disease (NAFLD) unrelated to obesity is highly prevalent in HFI patients [13]. Previously, Odievre et al. considered steatosis as a side effect of long-term strict avoidance of fructose due to an unbalanced diet containing an excess of fat [14]. Remarkably, as in healthy individuals, in HFI patients, fructose may be endogenously produced by the sorbitol-aldose reductase pathway, which can be activated in various physiological and not physiological circumstances, e.g., after a glucose-enriched meal, after administration of nephrotoxic drugs, during sepsis episodes or following cardiovascular surgery [15]. This endogenous production of fructose could partly explain the slight signs of liver disease present in most HFI patients in spite of FSS-free diet. Therefore, we support the idea that steatosis is a diet-unrelated marker of HFI, probably persisting for life [16]. However, none of our HFI patients showed signs of progressive liver disease in terms of cirrhosis or portal hypertension after a long period of observation. Maybe, as we have previously reported for a subset of children with Wilson’s disease, hypertransaminasemia can persist despite appropriate treatment in the absence of signs of progression of liver disease [17].

While some studies document phenotypic variability in HFI patients, the genotype–phenotype correlation has not been proved so far [18]. In this study, p.A175D homozygotes presented signs of mild liver disease more commonly than p.A150P homozygous patients. This finding can be explained considering that some variant enzymes retain residual activity [10,19]. As for other rare genetic conditions with wide allelic heterogeneity, genotype-phenotype analysis in HFI presents problems due to the difficulty in reaching adequate group size. Multicenter studies in larger cohorts of patients are desirable to confirm our data.

Recent data suggested a reversible transferrin hypoglycosylation in untreated HFI patients mimicking congenital disorders of glycosylation type I consistent with an increase of asialo- and disialo-, and a decrease of tetrasialo-transferrin [6]. The inhibition of phosphomannose isomerase by fructose 1-phosphate in the liver, kidney, and intestine, was postulated to be the mechanism of carbohydrate-deficient transferrin (CDT) in HFI [7]. It was also suggested that the introduction of a FSS-free diet normalizes or significantly decreases CDT levels [8]. Notably, evaluation of transferrin isoforms showed an altered profile in 23.7% of our HFI patients despite they were on long-term dietary treatment. In this study, we were not able to identify a safe threshold value of daily fructose intake. Nevertheless, we found a linear correlation between fructose intake and serum DST% and TST/DST. Therefore, serum CDT determination could be considered a good tool to monitor FSS intake and we could suppose that normal CDT profile is the desired therapeutic target in HFI patients. Furthermore, CDT profile could be useful to suggest maximum fructose daily intake tolerability of each HFI patient for personalized diet therapy.

## 5. Conclusions

In conclusion, our analysis reveals that HFI patients on a long-term FSS-free diet are asymptomatic, in good clinical conditions and without liver damage progression. Nevertheless, most HFI patients show mild signs of liver injury, i.e., increased ALT level and US abnormalities, throughout long-term follow-up. These alterations do not correlate with differences in residual dietary fructose intake, but they seem to be more severe in patients with specific genotypes. Although the pathogenetic mechanisms underlying the persistent abnormality of liver enzymes in HFI patients are unclear, physicians should be alert to this phenomenon to avoid expensive time-consuming diagnostic evaluations and ineffectual changes in diet. Although the HFI studied patients were generally adherent to the FSS-free diet, we observed that serum CDT profile determination was a reliable indicator of fructose intake. Therefore, the use of this tool could help in clinical practice to monitor fructose intake and eventually to prescribe a more rigorous and personalized diet in possible lower tolerant HFI patients.

## Figures and Tables

**Table 1:** Principal features of 48 hereditary fructose intolerance (HFI) patients and their daily fructose intake.

*n*	Sex	Age at Diagnosis (year)	Observation Period (year)	Symptoms at Onset	Liver Signs at Onset	Aldo B Mutation	Fructose Intake mg/day
1 *	M	0.3	4.9	-	B	p.A175D/p.A175D	0
2	M	0.9	7.6	1	A-B	p.A175D/p.A175D	0
3	F	0.6	21.1	2-4	A-B	p.A175D/p.Y204 *	165
4	F	1.1	14.9	1	A-B	p.A175D/p.A175D	223
5	M	0.8	9.7	-	A-B	p.Y204 */p.Y204 *	354
6*	M	0.8	13.9	2-3-4	A-B	p.A150P/p.A175D	223
7	M	2	14.6	4	A-B	p.A150P/p.A175D	223
8	F	0.6	19.2	1	A-B	p.A175DP/p.A175D	137
9	M	1	14.2	2	B	p.A150P/p.A150P	67
10	M	0.9	6.6	2-4	-	p.A150P/p.A175D	35
11	M	0.9	10.5	1	A-B	p.A150P/p.A175D	723
12	F	1.5	11.9	2-4	A	p.A150P/p.A150P	223
13	F	2.2	11.7	1	A-B	p.A150P/p.A150P	223
14 *	F	0	7.2	-	A-B	p.A150P/p.A150P	91
15	M	2.2	19.8	1	A-B	p.N120Kfs */p.N120Kfs *	91
16	M	1	4.3	1	A-B	p.A175D/p.L289Ffs *	45
17	F	3	16.5	1	A-B	p.L257P/p.N335K	40
18	F	18.5	4.5	1-4	B	p.A150P/p.A150P	104
19 *	M	0	17	1-2-3	B	p.A150P/p.A150P	253
20	F	4	18.2	1	-	p.A175D/-?	270
21	M	1	6.6	1	A-B	p.A150P/p.A150P	160
22	F	3	13.2	1	B	p.A175D/p.A175D	163
23 *	F	0	9.8	-	B	p.Y204 */p.Y204 *	154
24	F	0.5	16.3	1	B	p.A175D/p.Y204 *	109
25 *	M	0	12.1	-	-	p.A150P/p.A175D	223
26	M	2.1	4.3	1	A-B	p.A175D/p.A175D	46
27	F	0.6	6.9	1-3	A-B	p.A150P/p.A150P	91
28	M	0.7	6.3	4	A-B	p.A150P/p.Y204 *	325
29	F	10.8	15.2	-	B	p.A150P/p.Y204 *	198
30	F	1	11.4	1	B	p.A150P/p.A175D	247
31	F	4.7	5.6	1-2	-	p.A150P/p.A175D	91
32 *	M	0.2	8.6	-	-	p.A150P/p.A175D	91
33	F	0.5	4.2	2-4	-	p.A150P/p.A175D	0
34	F	0.3	6.7	2-4	-	p.Y204 */p.N335K	46
35	F	0.3	3.2	2-4	B	p.A150P/p.A150P	182
36	M	0.5	24.5	1-2-3	-	p.A150P/p.A150P	650
37	F	12	3.5	2	-	p.Y204 */p.Y204 *	110
38	F	7.5	4	2	-	p.A150P/p.A150P	91
39	M	5	4.1	1	A-B	p.A150P/p.A175D	6
40	M	1.3	5	4	A-B	p.A150P/c.1_624del	181
41	M	4	1.8	1	B	p.A150P/p.A150P	46
42	M	0.7	16.9	-	A-B	p.A150P/p.A150P	137
43	M	0.7	14.8	-	B	p.A150P/p.A150P	199
44	F	8	4	2	-	p.A175D/p.L289Ffs *	235
45	F	2	10.2	1	A-B	p.A150P/p.A175D	434
46	M	0.7	11.4	1-2-4	A-B	p.A175D/p.L229P	91
47	F	3	10.2	1	-	p.A150P/p.N120Kfs *	110
48	F	5.5	6.2	1	A-B	p.A175D/p.N335K	206

* older sibling affected. 1 sweet aversion; 2 vomit; 3 diarrhea, 4 hypoglycemia; A hypertransaminasemia, B US diagnosis of liver steatosis. M = male, F = female.

**Table 2:** ALT levels in 48 HFI patients distinguished in HFI patients with (H-group) and without (nH-group) persistent hypertransaminasemia, from diagnosis to the end of follow-up.

Time of Evaluation	ALT (U/L) Mean ± DS		*p*-Value
	H-Group	nH-Group	
T0	117 ± 115	61 ± 60	0.02
T1	91 ± 54	35 ± 26	<0.0001
T2	83 ± 44	28 ± 19	<0.0001
T3	70 ± 30	27 ± 16	<0.0001
T4	74 ± 38	24 ± 11	<0.0001
T5	52 ± 33	21 ± 8	<0.0001
T6	47 ± 26	21 ± 8	<0.0001
T7	85 ± 34	22 ± 8	<0.0001

T0: time of diagnosis; T1: 6 months; T2: 12 months; T3: 18 months; T4: 24 months; T5: 36 months; T6: 48 months; T7: end of follow-up.

**Table 3:** Other laboratory parameters in 48 HFI patients distinguished in HFI patients with (H-group) and without (nH-group) persistent hypertransaminasemia, at diagnosis and at the end of follow-up. T0: time of diagnosis; T7: end of follow-up.

	Time of Evaluation	H-Group	nH-Group	*p*-Value
GGT IU/L	T0	26.1 ± 16.5	18.3 ± 12.2	0.06
	T7	22 ± 11.2	17.6 ± 8.5	0.12
*p*-value		0.38	0.79	
Total Protein g/dL	T0	6.97 ± 0.79	6.89 ± 0.75	0.74
	T7	6.92 ± 0.62	6.98 ± 0.49	0.72
*p*-value		0.83	0.58	
Total Bilirubin mg/dL	T0	0.37 ± 0.18	0.41 ± 0.19	0.5
	T7	0.35 ± 0.18	0.4 ± 0.21	0.4
*p*-value		0.74	0.84	
Glucose mg/dL	T0	77.6 ± 9.5	73.2 ± 15.4	0.28
	T7	74.6 ± 10.7	78.7 ± 9	0.15
*p*-value		0.38	0.09	
